# Research on mood stabilizers in India

**DOI:** 10.4103/0019-5545.69265

**Published:** 2010-01

**Authors:** Ajit Avasthi, Sandeep Grover, Munish Aggarwal

**Affiliations:** Department of Psychiatry, Postgraduate Institute of Medical Education and Research, Chandigarh, India

**Keywords:** India, Mood stabilizers, Research

## Abstract

Mood stabilizers have revolutionized the treatment of bipolar affective disorders. We review data originating from India in the form of efficacy, effectiveness, usefulness, safety and tolerability of mood stabilizers. Data is mainly available for the usefulness and side-effects of lithium. A few studies in recent times have evaluated the usefulness of carbamazepine, valproate, atypical antipsychotics and verapamil. Occasional studies have compared two mood stabilizers. Data for long term efficacy and safety is conspicuously lacking.

## INTRODUCTION

Psychopharmacology has revolutionized the understanding and treatment of major mental disorders. With the help of psychopharmacological agents, not only is the neurobiology of various psychiatric disorders being understood, but effective treatments have improved relapse rates, symptom free period, significantly improved the quality of life of patients and have reduced the burden experienced by patients and their families.

Prior to lithium, typical antipsychotics and electroconvulsive therapy was used for management of bipolar disorders. However, over the years many drugs have been evaluated as mood stabilizers and have been shown to be efficacious, although the definition of a mood stabilizer is not yet settled.

Psychopharmacological research in India regarding mood stabilizers has lagged behind the data from the West. However, there has been a shift of research from mere case series to attempts at multicentric double blind controlled trials. In this review, we would review data on mood stabilizers originating from India on various mood stabilizers. The review shall focus on the research published in Indian Journal of Psychiatry and studies reported in PubMed indexed journals.

## MOOD STABILIZERS

Amongst various mood stabilizers now available in India, lithium has been the most researched of all. There are a few studies on other mood stabilizers such as carbamazepine and sodium valproate.

### Lithium

After its introduction in India in the late 1960s, lithium aroused a lot of research interest in the 1970s and 80s, with most of the research revolving around open trials to see its usefulness in various disorders (mainly mood disorders) and its side-effect.

### Effectiveness in mood disorders

The mood stabilizing property of lithium has led Indian researchers to see its effects in affective disorders. In their earliest work on role of lithium on mood disorders, Dube *et al*.[1] in an uncontrolled trial of lithium in 20 hypomanic patients found that 95% patients showed significant improvement. There have been other studies to see the effectiveness of lithium in mood disorders.[2–8] Most of these studies have been open label, non-controlled, with assessment period varying from one month to 10 years and have sparingly used assessment scales. Lithium has been found useful in treating acute episodes, reducing number of episodes, duration and intensity of episodes, behavior and suicidal ideation.[3,5,6,8] Studies have also looked for the socio-clinical correlates which influence the effectiveness of lithium. It has been shown that effectiveness of lithium is influenced by age and sex[4] and good response is predicted by lesser number of episodes prior to initiation of lithium therapy, long duration of illness,[4] and presence of family history of affective disorder.[2,5] Patients with rapid cycling mood disorder and unipolar disorder were seen to respond poorly.[2,4,9] Studies have also evaluated the utility of single dose vs. divided dose of lithium in prophylaxis of mood disorders. One study concluded that single daily dose was more useful in reducing number of affective episodes than divided doses[10] whereas another retrospective case review concluded that there was no difference in the efficacy and adverse effect in patients with once daily lithium versus lithium in divided doses.[11] In an effort to find effectiveness of lithium in mood disorders in children, Khandelwal *et al*.[12] found that lithium in dose of 750-1000 mg/ day (serum lithium of 0.6-1.2 mEq/L) was useful in acute control and chronic prophylaxis. Despite a good number of studies, most of these studies are limited in terms of small number of patients, short duration of follow up and neglect of important variables such as quality of life etc. Only one study from PGIMER, Chandigarh, evaluated the quality of life (QOL) of subjects with bipolar disorder stabilized on lithium prophylaxis using World Health Organization (WHO) Quality of Life-Bref (WHOQOL-BREF) and Quality of Life Enjoyment and Satisfaction Questionnaire (Q-LES-Q) along with factors that contribute or influence QOL and compared the same with schizophrenia and healthy control subjects. It was found that compared to the schizophrenia group, the bipolar group had significantly better QOL in all the domains of Q-LES-Q and the domains of general well-being, physical health and psychological health of the WHOQOL-Bref. The bipolar group had similar QOL scores in all other domains and higher scores in leisure time activity domain of Q-LES-Q, in comparison to the healthy group. These findings suggest that lithium contributes significantly to improvement in QOL in subjects with bipolar disorders.[13]

With regard to early onset bipolar disorder a retrospective chart review found that lithium was the most commonly used mood stabilizer, followed by valproate. However, during the follow-up period of three to 56 months it was seen that 28% of subjects relapsed despite being on apparently adequate doses of lithium.[14]

With regard to organic mania, a recent case report by Loganathan *et al*.[15] reported usefulness of lithium in treatment of mania in subjects with Wilson’s disease.

### Predictor of response to lithium

Sampath *et al*. (1980)[16] determined RBC/plasma lithium ratio in 35 manic patients who were on lithium for three months and determined its relation to response. It was found that ratio was higher in responders than non- responders. Similarly, Pradhan and Channabasavanna[17] examined the relation of intracellular Na^+^, K^+^, Li^+^ to extra cellular concentration of these ions in a group of 22 patients of bipolar affective disorder who were divided into responders and non-responders. No significant difference between two groups was found as regards to these values.

### Useful in schizophrenia and other disorders

In a double blind placebo controlled cross over trial between lithium and chlorpromazine lasting for four weeks, Dube and Sethi[18] reported that lithium was not found to be superior to chlorpromazine in severity and global improvement but selective effectiveness of lithium was seen in controlling hyperactivity, excitement, hostility and emotional withdrawal symptoms in schizophrenia and based on this the authors recommended use of lithium as adjuvant for specific symptoms. However, one study showed that lithium was ineffective in chronic hypochondriacal and schizoaffective disorders.[19]

Studies have also showed that lithium is useful in management of aggression in patients of various diagnostic categories who were non-responsive to antipsychotics, electroconvulsive therapy, antiepileptics and sedatives. Authors recommended use of lithium in dose of 500- 750 mg/day (Serum lithium 0.75-1.2 mEq/L) in patients with unspecified aggression.[20] Lithium has also been used successfully for treatment of Kleine-Levin Syndrome,[21] Ganser syndrome[22] and Neuroacanthocytosis.[23]

### Research on serum lithium levels and related issues

Attempts have been made to estimate saliva levels and see their correlation with serum lithium levels with conflicting results. Prakash *et al*.[24] investigated equal number of serum and saliva samples for lithium level estimation and reported high correlation between the two. On the other hand, in a methodologically and statistically strong study undertaken by Natraj and Bhat,[25] lithium levels in saliva were reported to be an unreliable indicator of serum lithium levels. Pandey *et al*.[26] compared the serum lithium levels with use of single equivalent dose of sustained release lithium preparation and plain lithium preparation in 18 healthy volunteers. Serum lithium levels were estimated at four-hourly intervals. With plain lithium preparation majority of patients achieved serum lithium within therapeutic range but only half of patients with sustained release preparations achieved the therapeutic range. Further, the standard deviations of levels of lithium were more with sustained release suggesting high fluctuations in serum levels. The study is important in the context that more and more practitioners tend to use sustained release preparation, which might lead on to fluctuation in serum level which practitioners may not be aware of. Based on observation of widely fluctuating serum levels with respect to time and quality control, Kuruvilla *et al*.[27] observed that it was essential to run a quality control sample with each test sample as an independent check prior to estimation. It was also pointed out that similar equivalent result can be obtained whether blood sample is analyzed within six hours or 5-6 days. Similar findings were reported by Khandelwal *et al*.[28] who based on sample analysis of two groups of patients (in one sample stored at room temperature for 6 days and other stored at −4°C for 8 days) concluded that lithium levels remained same irrespective of time of analysis. This observation may be important keeping in view limited number of laboratories in India and time spent on transporting samples. Kuruvilla and Shaji[29] found that 24 hours blood levels of lithium after test dose were not reliable enough to predict therapeutic serum lithium levels. In a recent study significantly high variability of lithium levels in different months of the year was reported and authors suggested that frequent serum level monitoring and oral lithium dose adjustment needs to be done to prevent situations of toxicity and lack of efficacy.[30] In a case series (N = 3), Tripathi *et al*.[31] reported that when compared to standard normal diet bioavailability of lithium is reduced by high fat food.

### Side-effects of lithium

Research on side-effects of lithium has been focused on general side-effects and specific side-effects on various systems.

### General

Venkoba Rao *et al*.[32] examined side-effects to lithium (dose 750-1000 mg/day, serum lithium between 0.6-1.2 m Eq/L) in patients of mood disorders who were receiving lithium for three months and reported that nearly 81% patients had at least one side-effect with 14% having more than one side-effect. Common side-effects reported were tremors, polyuria, memory deficits, excessive salivation and gastrointestinal side-effects. In another comparative study which evaluated the side-effects of lithium, carbamazepine and haloperidol in acute mania, Trivedi *et al*.[33] reported that though total side-effects were not much different between the three groups, the rate of amelioration was best for lithium and least for haloperidol, indicating lithium to be better tolerated.

### Cognitive functions

One study compared the cognitive function of 34 bipolar patients on lithium (serum lithium between 0.6-1.2 mEq/L) with 30 matched controls. There was no difference on cognitive testing as measured by Bhatia Battery test, PGI memory scale and Bender Gestalt test. However, on self assessment of cognitive functions, patients experienced feeling of subjective cognitive impairment.[34] Researchers have also used animal models to study the effects of lithium on memory functions. In an experimental design on 30 albino rats, Sethi *et al*.[35] performed brightness discrimination and observed reaction time, positive response during relearning and relearning index; and found that mice exposed to chronic administration of lithium had reduction in size of memory trace laid during previous learning. Further, lithium interfered with ability to perform learned behavior and long term memory.

### Neurological

Residual neurological side-effects after lithium toxicity in the form of dysarthria, nystagmus and generalized cerebellar damage have been reported.[36–40] Andrade *et al*.[41] also reported that lithium neurotoxicity can occur with therapeutic lithium levels. In another retrospective chart review of patients with lithium toxicity, Kumar *et al*.[42] reported that toxicity even occurs with therapeutic lithium levels and is commonly seen in subjects receiving antipsychotics along with lithium and cerebellar symptoms are the most common presentation, which occur at significantly lesser serum levels. Chakrabarti and Chand[43] reported a case of tardive dystonia with lithium.

### Renal

Five controlled studies have examined renal side-effects of lithium in humans.[32,44–47] In comparison to age and gender matched controls, it has been shown that patients on lithium for variable duration have been found to have anti- diuretic hormone resistant concentration deficit resulting in polyuria, impaired creatinine clearances, low renal tubular H^+^ excretion, normal glomerular filtration rate (GFR) and renal tubular acidification. It has also been shown that serum lithium levels correlate positively with daily urine volume and negatively with urine specific gravity. Studies have also shown that there is no relationship between total amount of lithium consumed or duration of treatment with renal impairments.[32,44,46] A recent study showed that there was no difference in GFR between affective disorders patients on lithium and normal controls.[48] Another study reported lack of deterioration in renal functions of subjects on lithium for 1 year.[49] In one study renal biopsy of patients on lithium showed non-specific abnormalities of glomerular hypercellularity, cloudy swelling of tubular epithelium and interstitial fibrosis. However, these findings had no correlation with renal functioning and duration of lithium treatment.[45] Despite the above conclusive findings, most of the studies mentioned have a small sample size, are cross sectional and poorly controlled.

### Thyroid functions

Despite well-documented adverse effects of lithium on thyroid functions and good quality research from West, there has been little enthusiasm to study thyroid functions in Indian subjects. In an uncontrolled study which included 40 patients with bipolar disorder on prophylactic lithium with serum lithium of 0.83 (SD 0.20 mEq/L), Kuruvilla *et al*.[50] assessed thyroid functions at baseline and after two years and didn’t find any significant difference in total T_4_, free thyroid index or TSH. Srivastava *et al*.[51] compared thyroid hormone level of 30 patients with bipolar disorder on lithium (6-24 months) and with healthy controls and found an increase in the level of TSH (more than the normal range) in 20% of subjects, normal T_3_ levels and decreased T_4_ levels in 13% of subjects with bipolar disorder. The authors noted that high dose and high serum lithium levels increase the possibility of reduction of thyroid function status. In a case report Jayesh[52] reported clinical hypothyroidism in a male patient on lithium therapy for 12 months, which persisted despite discontinuation of lithium.

In a study from PGIMER, Chandigarh, Deodhar *et al*.[53] compared the thyroid functions of 24 healthy controls and 132 affective disorder subjects. They found that lithium treatment induced sub-clinical hypothyroidism in 51/132 (39%) of patients and the levels of T4 declined significantly (*P* < 0.05) with lithium treatment ranging from 61 to 240 months as compared to the corresponding values in the control group.

### Cardiovascular

Venkoba Rao and Hariharsubramaniam[54] examined ECG changes in patients on lithium for 1-43 months (dose 900-1500 mg/day, serum lithium 0.8-1.2 mEq/L) and reported slow sinus rhythm, prolongation of intraventricular conduction and interference with repolarization. Longer duration of therapy and age were found to influence the ECG changes. Based on their finding the authors recommended occasional ECG monitoring in elderly patients on long-term lithium therapy. Kurpad *et al*.[55] reported a case of ventricular ectopic in a patient on lithium. In a recent case report, Praharaj *et al*.[56] reported bradycardia with lithium at therapeutic doses in a case of bipolar disorder with bilateral thenar hypoplasia, which resembles a milder variety of Holt-Oram syndrome.

### Electrolyte Disturbance

Srinivasan *et al*.[57] assessed Mg^+2^, Ca^+2^ levels in 17 patients of affective disorder, on lithium and followed them prospectively and observed increase of total serum Mg^+2^, which was more so in patients who relapsed and the authors pointed at deficiency of Mg^+2^ at cellular level as a probable cause of episodes.

### Tilak effect

Parvathi Devi and Rao[58] labeled the stimulatory action of lithium on pineal gland as “Tilak effect.” Same groups of authors researched effects of lithium on adrenal cortex in an experimental group of mice.[59]

### Pregnancy

In a case report Grover and Gupta[60] reported congenital malformation in the form of anencephaly with use of lithium in first trimester. In another case report, still birth was reported by Khandelwal *et al*.[61] with the use of lithium during pregnancy.

### Psychiatric Side-effects

In a case report, abrupt withdrawal of lithium in a patient with recurrent depressive disorder led to development of hypomania.[62]

### Carbamazepine

#### Usefulness

In contrast to lithium there has been little research on use of carbamazepine in psychiatric disorders from India. In a controlled trial, Sethi *et al*.[63] recruited 16 patients with affective disorder (unipolar or bipolar); eight were put on carbamazepine (1000-1600 mg/day) and another eight on either chlorpromazine or imipramine. Patients in the depressive phase were given carbamazepine or imipramine and patients in manic phase were given either carbamazepine or chlorpromazine. Six out of eight patients on carbamazepine showed moderate to marked improvement in symptoms after 4 weeks of treatment. One patient showed 50% improvement and another patient on carbamazepine discontinued the therapy due to marked side-effects. Desai *et al*.[64] reported a case of mania treated with combination of lithium and carbamazepine.

Based on the kindling hypothesis, Das *et al*.[65] divided the subjects of bipolar disorders as kindlers (as patients with 3 or more affective episodes in ≤1 year) and non-kindlers (as patients with at least 3 episodes in the past with inter episodic period of more than 1 year) and evaluated the response to carbamazepine (mean dose- 718 ± 109 mg/day) over a period of six months and reported no significant difference between the two groups based on sex, past history of substance intake, type of mood (irritable versus elated), number of relapses during the follow-up and improvement on carbamazepine. Sadanandan and Anand[66] reported three patients with epilepsy and interictal hyper-religiosity who responded well to become asymptomatic with carbamazepine. In a case report, Patkar *et al*.[67] reported that carbamazepine may be effective in prepubertal lithium refractory rapid cycling subjects.

#### Side-effects

A case series suggested that carbamazepine (600 mg/day) can lead to worsening of psychotic symptoms in toxic range.[68]

### Valproate

#### Usefulness

Among the three mood stabilizers, valproate has been used most recently for treatment of mood disorders. Some of the recent studies have compared the efficacy/effectiveness of valproate with other mood stabilizers.[69–74] A few case series have shown effectiveness of intravenous valproate in management of acute mania without any serious adverse effects.[75,76]

#### Usefulness in other conditions

Chadda *et al*.[77] evaluated the usefulness of sodium valproate (1200 mg/day) for four weeks in tardive-dyskinesia and showed statistically significant improvement after two weeks which persisted at four-week assessment. In a case report, valproate has also been found to be useful in management of menstrual psychosis.[78]

#### Side-effects

In a recent case series, Pradeep[79] presented three cases of delirium with hyperammonemia in subjects receiving valproate monotherapy.

### Atypical Antipsychotics

In a three-week randomized, double-blind trial, Khanna *et al*.[80] included subjects of bipolar I disorder with mania or mixed episode as per DSM-IV diagnosis, who were more than 18 years old, and had a baseline Young Mania Rating Score of more than 20. The subjects were admitted to hospital and then randomly assigned in equal numbers to receive placebo or 1-6 mg of risperidone. Randomization was stratified by the presence or absence of psychotic features at baseline, manic or mixed episode and by treatment centre. Risperidone was received by 146 patients and placebo by 144. Their mean baseline YMRS score was 37.2. Treatment efficacy was determined primarily by the change from baseline to end-point in mean total scores on the Young Mania Rating Scores. At the end of three weeks, significantly greater improvement were observed with risperidone than with placebo at weeks 1 and 2 and at end-point (total YMRS: *P* < 0.01). The mean YMRS score of 37.2 at baseline reduced to 14.5 at the end of the trial in the risperidone group and this was significantly better than the placebo group. Extrapyramidal side-effects were reported by 35% of subjects in the risperidone group (compared to 6% in the placebo group) and these were the most frequently reported adverse events in the risperidone group. Another side-effect which was reported by more than 10% of subjects was insomnia; however there was no difference between the two groups on the incidence of the same.

### Studies comparing various mood stabilizers

Few studies have compared the efficacy/effectiveness of various mood stabilizers in affective disorders. In a randomized controlled trial of four weeks duration, Prakash and Bharath[69] compared the efficacy of valproate (N = 10; 600-1200 mg/day) and lithium (N = 11; 900- 1500 mg/day) in the treatment of acute mania and found that valproate was as efficacious as lithium in controlling the acute manic episode. Of the 10 patients randomized to valproate, 80% responded to 600 to 1200 mg of sodium valproate within two to three weeks indicated by a 50% reduction in total scores in Beigel’s Mania Rating Scale (BMRS). Subjects in both the groups required equal doses of adjuvant benzodiazepines. Side-effects of valproate were similar to lithium except that 1 patient in valproate group developed hepatitis. Another study compared antimanic efficacy of carbamazepine (started at 20 mg/kg body weight per day and titrated as per requirement) with valproic acid (started at 20 mg/kg body weight per day and titrated as per requirement) and reported that valproic acid was more efficacious than carbamazepine (CBZ).[70] Of the valproic acid treated patients, 73% showed a favorable clinical response while 53% of the patients on carbamazepine responded favourably. Using the intent-to-treat analysis, it was seen that valproic acid led to significant reduction in YMRS total scores after week 1 while the carbamazepine group showed significant reduction after week 2.

In the primary efficacy analysis, valproate group experienced significantly greater mean improvement in Young Mania Rating Scale total score than the carbamazepine group. In the CBZ group, significantly more patients required rescue medication during the week 2 and the requirement was quantitatively more as compared to the valproic acid group.[70] In a recent 12 week, randomized, double-blind pilot study involving 60 subjects diagnosed with acute mania (DSM-IV), Kakkar *et al*.[71] compared the efficacy and safety of oxcarbazepine (1,000-2,400 mg/day) and divalproex (750-2,000 mg/day) in the treatment of acute mania. In both the groups, the authors found comparable improvement in mean YMRS scores including the mean total scores as well as percentage fall from baseline. There were no significant differences between treatments in the rates of symptomatic mania remission (90% in divalproex and 80% in oxcarbazepine group) and subsequent relapse. Median time taken to symptomatic remission was 56 days in divalproex group while it was 70 days in the oxcarbazepine group (*P* = 0.123).

A significantly greater number of patients in divalproex group experienced one or more adverse drug events as compared to patients in the oxcarbazepine group (66.7% versus 30%, *P* <; 0.01). In a 1 week parallel group randomized comparative prospective study trial, Solanki *et al*.[72] evaluated the effectiveness of injectable sodium valproate (1500-2000 mg/day given in divided doses by IV infusion with 500 cc of 5% distilled water) in controlling the acute mania (N = 60) and showed that there was substantial improvement in YMRS scores and valproate was significantly better than risperidone. Of the 30 subjects randomized to valproate, 18 showed more than 50% reduction in Young Mania rating scale (YMRS) scores, six showed minimal improvement (less than 25% reduction in YMRS score), four showed no changes and two patients became worse at the end of the trial (because of psychotic features). No serious adverse effects were seen in any patient on valproate and the adverse effects which were noted were of mild intensity and included marginal (benign) elevation of liver enzymes (SGOT and SGPT) in 24 patients, flatulence in one patient, diarrhea in two patients, vomiting in 3 patients, nausea in 5 patients and thrombophlebitis in one patient. Findings of this study suggest that injectable valproate may be a good option in management of acute mania. Another study compared lithium (N =25) and verapamil (N = 25) in a four-week double-blind randomized controlled trial in the management of acute mania. The subjects were rated on Bech-Raefelson Mania Rating Scale and Young-Mania Rating scale. At the end of four weeks the authors found that lithium and verapamil have equal efficacy.[73]

In a naturalistic follow-up study of four years, of subjects presenting with first episode mania (N = 51), Khess *et al*.[74] compared the effectiveness of lithium, carbamazepine, valproate and antipsychotic in preventing the recurrence of symptoms and found that patients receiving lithium had the best outcome with only 30.77% having recurrence compared to 70.65% subjects on other drugs and 100% on no drugs. Patients with family history and poor compliance had greater number of relapses and late age of onset and concurrent use of substance led to greater number of admissions.

### Mood Stabilizer polypharmacy

#### Usefulness

In an open label six-month prospective randomized controlled trial, Gangadhar *et al*.[81] compared the efficacy of lithium alone (900 mg/day; serum levels of 0.8- 1.2) with lithium (900 mg/day; serum levels of 0.8-1.2) plus carbamazepine (200 mg BD) and reported statistically non-significant trend favoring the combination therapy and suggested that addition of carbamazepine hastens recovery and leads to less frequent use of emergency sedation without any increase in side-effects. The findings of Gangadhar *et al*.[81] are also supported by a case report in which the authors reported that lithium (1500 mg/ day) along with carbamazepine (600 mg/day) led to marked clinical improvement with sustained recovery in a patient who showed poor response to lithium, neuroleptics and ECT and developed severe extrapyramidal side-effects restricting the use of neuroleptics in high doses.[64]

#### Side-effects due to use of polypharmacy

Various case reports have suggested that use of multiple medications in subjects with affective disorders can lead to side-effects like Steven Johnson syndrome.[82–85] Case reports also suggest development of neuroleptic malignant syndrome in young subjects when lithium is combined with olanzapine[86] or haloperidol and carbamazepine.[87]

## CONCLUSION AND FUTURE DIRECTIONS

Most of the literature on mood stabilizers pertains to lithium. Studies have found it to be useful in Indian patients for management of bipolar disorders. In recent times, studies have also compared valproate with lithium and have reported it to be as effective as lithium and more effective than risperidone in management of acute mania. Valproate has also been reported to be better than carbamazepine but equally efficacious to oxcarbazepine in management of acute mania. One double blind randomized controlled trial has also demonstrated risperidone to be better than placebo in the management of acute mania. Studies have also shown that addition of carbamazepine to lithium may be useful in management of acute mania.

However, major limitations of the research have been small sample size and lack of multicentric studies. There are no studies which have evaluated the efficacy or effectiveness of various mood stabilizers in the management of bipolar depression. There is dearth of data with regard to usefulness of various medications for prophylaxis. There is an urgent need to conduct multicentric studies.
